# Mannitol Utilisation is Required for Protection of *Staphylococcus aureus* from Human Skin Antimicrobial Fatty Acids

**DOI:** 10.1371/journal.pone.0067698

**Published:** 2013-07-04

**Authors:** John G. Kenny, Josephine Moran, Stacey L. Kolar, Alexander Ulanov, Zhong Li, Lindsey N. Shaw, Elisabet Josefsson, Malcolm J. Horsburgh

**Affiliations:** 1 Institute of Integrative Biology, University of Liverpool, Liverpool, Merseyside, United Kingdom; 2 Department of Cell Biology, Microbiology & Molecular Biology, University of South Florida, Tampa, Florida, United States of America; 3 Roy J. Carver Biotechnology Center, University of Illinois, Urbana-Champaign, Illinois, United States of America; 4 Department of Rheumatology and Inflammation Research, University of Gothenburg, Göteborg, Sweden; National Institutes of Health, United States of America

## Abstract

Mannitol (Mtl) fermentation, with the subsequent production of acid, is a species signature of *Staphylococcus aureus*, and discriminates it from most other members of the genus. Inactivation of the gene *mtlD,* encoding Mtl-1-P dehydrogenase was found to markedly reduce survival in the presence of the antimicrobial fatty acid, linoleic acid. We demonstrate that the sugar alcohol has a potentiating action for this membrane-acting antimicrobial. Analysis of cellular metabolites revealed that, during exponential growth, the *mtlD* mutant accumulated high levels of Mtl and Mtl-P. The latter metabolite was not detected in its isogenic parent strain or a deletion mutant of the entire *mtlABFD* operon. In addition, the *mtlD* mutant strain exhibited a decreased MIC for H_2_O_2_, however virulence was unaffected in a model of septic arthritis.

## Introduction

*S. aureus* is a common skin and soft tissue pathogen capable of causing more severe infections including sepsis, osteomyelitis, and endocarditis [1]. The range of infections is due to a multitude of encoded virulence factors and nasopharyngeal carriage is frequent and a risk factor [2], [3]. The spread of antibiotic-resistant strains and the emergence of community-acquired MRSA have increased the impact of *S. aureus* on public health and it has necessitated the development of new therapeutics plus a better understanding of transmission and skin survival [4].

Several different barrier functions are proposed to retard the survival of *S. aureus* on human skin, these include the antimicrobial peptides cathelicidin LL-37 and human β-defensin 2, as well as dermicidin, psoriasin, RNase3 and RNase7. One focus for study of survival is the antimicrobial activity of long chain (typically C≥16) unsaturated free fatty acids that generate the acid mantle on skin [5], [6], [7], [8], [9]. These antimicrobial fatty acids (AFAs) are components of the innate immune system that function on skin and in abscesses [9], [10], [11], [12], [13], [14], [15], [16], [17], [18]. The amphipathic properties of AFAs are proposed to disrupt membrane function by altering permeability and fluidity and this is supported by transcriptional analyses of linoleic acid-treated *S. aureus* [6]. Cells exposed to sub-inhibitory concentrations of linoleic acid respond by upregulating transcription of genes encoding capsule, peptidoglycan and carotenoid biosynthetic enzymes and pathways for stress resistance [6]; glycolysis and fermentation pathway genes are concomitantly upregulated. In *S. aureus* protection against AFAs is afforded by reducing cell surface hydrophobicity [6], [7], [19] and the described transcriptional upregulation of cell surface components is proposed to mediate this effect [6]. The transcript encoding the cell surface protein SasF is upregulated >30 fold after addition of linoleic acid and inactivation of the gene decreases survival, but not via detectable changes to surface hydrophobicity [6]. In contrast, cell wall teichoic acid (WTA) and the iron-regulated surface protein IsdA increase survival from AFAs by decreasing surface hydrophobicity [7], [19]. Inhibitory concentrations of AFAs cause leakage of proteins and inhibit respiration [20], [21], [22], [23].

In this study, extended screening of *S. aureus* mutants with reduced survival from AFAs identified identical clones with defective mannitol (Mtl) metabolism. Since the capacity of staphylococci to ferment Mtl is most frequently associated with the pathogens *S. aureus*, *S. saprophyticus* and *S. haemolyticus*, we sought to determine the nature of the survival defect in the context of cellular resistance and virulence.

## Materials and Methods

### Bacterial Strains, Plasmid and Growth Conditions

Strains and plasmids used in this study are listed in Table 1. Bacteria were grown in brain heart infusion broth (BHI) (Lab M) at 37°C with shaking at 125 rpm, unless indicated otherwise. Mannitol broth contained: peptone 10 g l^−1^, Mtl 10 g l^−1^, beef extract 1 g l^−1^, NaCl 10 g l^−1^; fructose broth contained fructose in place of Mtl. Cultures were incubated at 37°C with shaking at 250 rpm and growth was monitored by measuring OD_600_. When included, antibiotics were added at the following concentrations: erythromycin, 5 µg ml^−1^; lincomycin, 25 µg ml^−1^; tetracycline, 5 µg ml^−1^, chloramphenicol 5 µg ml^−1^. Antibiotics were not included in comparative growth experiments.

**Table 1:** Strains and plasmids used in the study.

Strain or Plasmid	Features	Reference or Source
**Strains:**		
*E. coli:*		
Top10	F- *mcrAΔ(mrr-hsdRMS-mcrBC) Φ80lacZΔM15 ΔlacX74 recA1 araD139 Δ (ara leu) 7697 galU galK rpsL (strR) endA1 nupG*	Invitrogen
Liv1008	Top10-pMutin4-*mtlD*	This Study
Liv1011	Top10-pMutin4-*mtlABFD*	This Study
*S. aureus:*		
SH1000	Functional *rsbU* derivative of 8325-4 *rsbU*+	Lab strain
RN4220	Restriction-deficient strain	Lab strain
suvB24	SH1000 *mtlD::Tn917*	This Study
Liv772	Newman *mtlD::Tn917*	This Study
Liv1019	RN4220-pMutin4-*mtlD*	This Study
Liv1020	RN4220-pMutin4-*mtlABFD*	This Study
Liv1023	SH1000 *mtlD::tet*	This Study
Liv1024	SH1000 *mtlABFD::tet*	This Study
Liv1027	Newman *mtlD::tet*	This Study
Liv1028	Newman *mtlABFD::tet*	This Study
Liv1090	RN4220 *mtlD::tet* pMJH70	This Study
Liv1091	RN4220 *mtlD::tet* pMJH71	This Study
Liv1097	SH1000 *mtlABFD::tet* pMJH71	This Study
Liv1098	SH1000 *mtlD::tet* pMJH71	This Study
**Plasmids**		
pLTV1	Temperature sensitive plasmid harbouring Tn*917*	[24]
pMUTIN4	Insertional inactivation vector	[26]
pDG1513	pMTL22 derivative, tetracycline resistant	[25]
pSK5632	Low copy number shuttle plasmid	[27]
pMJH70	pSK5632 containing *mtlD*	This study
pMJH71	pSK5632 containing *mtlABFD*	This study

### Construction of *mtl* Mutants and Complementation Plasmids

Construction of *mtlD* and *mtlABFD* allelic replacement mutants was performed using methods described previously [24]. Amplification of *mtlD* for allelic replacement used upstream and downstream primer pairs, *mtlD*_*BamH*I CGACGGATCCGATGTTGATGGCAACACATC with *mtlD*_*Not*I ATAACTGCGGCCGCCAGCACCAAAGTGAACTGC and *mtlD*_*Kpn*I CCGGTACCTAGCCGATGAAATAATTG with *mtlD*_*EcoR*I ACATGAATTCAACTAATGACAAGGTTGC and for *mtlABFD* operon allelic replacement the primer pair *mtlA*_*BamH*I CGACGGATCCTAACTTCTGTATCTGTTTCTG and *mtlA*_*Not*I ATAACTGCGGCCGCTCTCTTCAGTTTGTGACATG. The downstream operon fragment was amplified using *mtlD*_*Kpn*I and *mtlD*_*EcoR*I. The tetracycline resistance gene (*tet*) was amplified from pDG1513 [25] followed by simultaneous cloning of *tet* disrupted alleles into pMUTIN4 [24], [26] and the resultant plasmids pJK1 and pJK2 containing the *mtlD*-*tet* and *mtlABFD*-*tet* inserts, respectively, were used to generate allelic replacement mutants in strain SH1000. Plasmids to complement the *mtl* mutations were made by ligating the *mtlABFD* operon, amplified using *mtlA*_*Sal*I ACGCGTCGACCGAACTTTCCCCCTTTCC and *mtlD*_*BamH*I ACGCGGATCCGAACTACTACATTATTACTGATTG or *mtlD*_*Sal*I with *mtlD*_*BamH*I. The amplicons and pSK5632 [27] were digested and ligated prior to directly transforming Liv1019 (RN4220 *mtlD*::*tet*), selecting for acid production on Mtl salt agar containing 5 µg ml^−1^ chloramphenicol. The selected plasmid, pMJH71, was purified and used to transform Liv1023 (SH1000 *mtlD*::*tet*) and Liv1024 (SH1000 *mtlABFD*::*tet*).

### Antimicrobial Fatty Acid Survival and MICs

An agar plate assay for AFA survival described previously [6] was used to measure comparative growth. Serial dilutions of the mutant strains were plated onto BHI agar containing millimolar concentrations of AFA, prior to viable counting. Minimum inhibitory concentrations (MIC) of AFAs were performed in 96 well plates using ethanol as a solvent.

### Zeta potential and Hexadecane Partitioning

Zeta potential was determined using electrophoretic light scattering (ELS) in which the velocity of charged particles under the influence of an applied electric field is measured by monitoring the frequency shift of the scattered light from the particles. Culture (∼800 µl) was injected into a capillary cell and measured using a Zetasizer Nano (Malvern Instruments) with the detector positioned at a 17° scattering angle. The data were analysed and interpreted using the associated software. All charges were recorded as the mean of 5 consecutive measurements. Hexadecane partitioning was performed as previously described [6].

### BioLog Phenotypic Arrays

BioLog phenotypic arrays were used to monitor growth of bacterial strains in 96 well plates under a wide range of conditions using redox levels within the growth media as a measure of bacterial growth [28]. Strains SH1000 or suvB24 were resuspended from BHI plates to a transmittance of 81% using a BioLog turbidometer then added to the appropriate inoculation fluid for each assay plate. Comprehensive details of the growth factors tested using these assay plates PM1-PM10 can be found at http://www.biolog.com/pdf/pm_lit/PM1-PM10.pdf. Following inoculation the array plates were incubated at 37°C and monitored for turbidity using the OmniLog plate reader at 30 min intervals over a 47 hour period. The assay was performed in triplicate and the mean values were used to compare growth.

### Metabolite Analysis

Four hour cultures of strains SH1000, Liv1023 (SH1000 *mtlD*::*tet*) and Liv1024 (SH1000 *mtlABFD*::*tet*) were harvested by centrifugation and washed 3 times in PBS, before being resuspended in 3 ml of PBS. Cells were lysed using a bead-beater for three 1 min intervals at 4°C, with chilling between breakages. Cytoplasmic fractions were centrifuged, and metabolic reactions quenched via the addition of methanol. Samples were then dried down and derivatized as described previously [29], [30] with the following modifications. Samples were incubated for 90 min at 50°C with 80 µl of methoxyamine hydrochloride in pyridine (20 mg ml^−1^) following a 60 min treatment at 50°C with 80 µl MSTFA. Five µl of an internal standard (C31 fatty acid) was added prior to trimethylsilylation, and sample volumes of 1 µL were injected with a split ratio of 7∶1. The GC-MS system consisted of an Agilent 7890A (Agilent Inc, Palo Alto, CA, USA) gas chromatograph, an Agilent 5975C mass selective detector and Agilent 7683B autosampler. Gas chromatography was performed on a 60 m HP-5MS column with 0.25 mm inner diameter and 0.25 µm film thickness (Agilent Inc, Palo Alto, CA, USA), and an injection temperature of 250°C. The interface was set to 250°C, and the ion source adjusted to 230°C. Helium carrier gas was set at a constant flow rate of 1.5 ml min^−1^. The temperature program was 5 min isothermal heating at 70°C, followed by an oven temperature increase of 5°C min^−1^ to 310°C, and a final 20 min at 310°C. The mass spectrometer was operated in positive electron impact mode (EI) at 69.9 eV ionization energy in a m/z 30–800 scan range. The spectra of all chromatogram peaks were compared with electron impact mass spectrum libraries NIST08 (NIST, MD, USA), WILEY08 (Palisade Corporation, NY, USA), and a custom library. To allow comparison between sample sets, all data were normalized to internal standards in each chromatogram, and the weights of each sample. The chromatograms and mass spectra were evaluated using the MSD ChemStation (Agilent, Palo Alto, CA, USA) and AMDIS (NIST, Gaithersburg, MD, USA) programs. The retention time and mass spectra were implemented within the AMDIS method formats. The resulting data from triplicate samples (with less than 10% variability) was analyzed using a t-test. Samples with a p<0.05 and greater than 2-fold variation were then analyzed using the MetPA enrichment pathway analysis web application (http://metpa.metabolomics.ca/) [31].

### Experimental Septic Arthritis

A previously described mouse model of septic arthritis was used to test the *in vivo* role of *mtlD* in virulence [32], [33]. Seven week female NMRI mice were obtained from Charles River Laboratories (Sulzfeld, Germany) and maintained in the animal facility of the Department of Rheumatology and Inflammation Research, University of Göteborg, Sweden. All mice were maintained according to the local ethic board animal husbandry standards. The mice were housed 10 to a cage under standard conditions of temperature and light and were fed standard laboratory chow and water *ad libitum*. Mice were inoculated in the tail vein with 0.2 ml of bacterial suspension cultured and bacteria in kidney abscesses were enumerated after 14 days as described previously [6]. Presented data represent CFU per kidney pair.

## Results

### Identification of a *mtlD* AFA Survival Mutant

**Figure 1:**
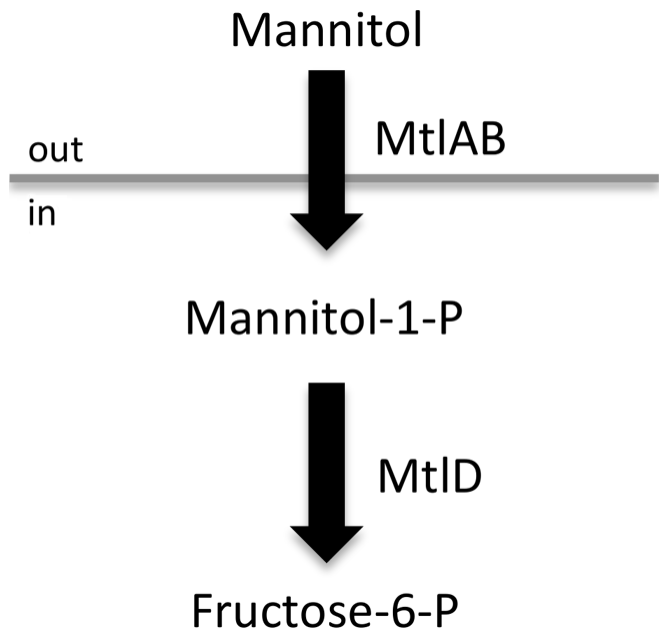
Mannitol uptake pathway in *S. aureus*. The *mtlABFD* operon encodes the Mtl-specific PTS (MtlAB) and the operon transcriptional repressor (MtlF); Mtl-1-P 5-dehydrogenase, encoded by *mtlD*, catalyses the conversion of Mtl-1-P to fructose-6-P which enters into the Embden-Meyerhoff and hexosemonophosphate glycolytic pathways.

**Figure 2:**
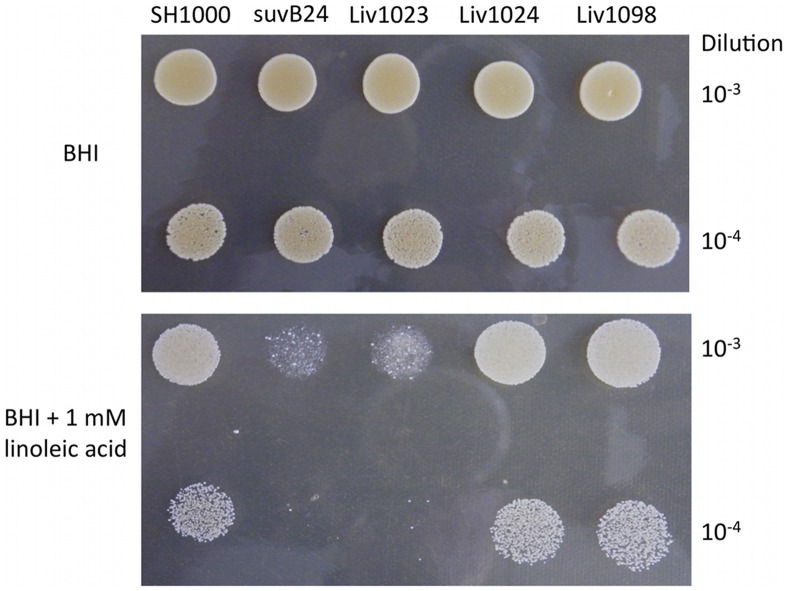
Comparative survival of *S. aureus* strains. Growth of dilutions from overnight cultures on BHI agar in the presence and absence of 1 mM linoleic acid. SuvB24 (SH1000 *mtlD*::Tn*917*) and Liv1023 (SH1000 *mtlD*::*tet*) displayed >500-fold reduced survival on linoleic acid relative to wild type (SH1000), Liv1024 (SH1000 *mtlABFD*::*tet*) and the complemented mutant strain Liv1098 (SH1000 *mtlD*::*tet* pMJH71).

A screen of *S. aureus* Tn*917* library transposants identified multiple clones with greatly reduced survival on BHI agar containing 1 mM linoleic acid (C_18∶2Δ9Δ12_), in addition to those mutants described previously [6]. DNA sequence determination by arbitrary-primed PCR [6] revealed these clones were identical, with Tn*917* inserted in *mtlD* at nucleotide position 317/1107. The *mtlABFD* operon encodes the Mtl-specific phosphotransferase system (PTS) trasnsporter (MtlAB) and the operon transcriptional repressor (MtlF); Mtl-1-P 5-dehydrogenase, encoded by *mtlD*, catalyses the conversion of Mtl-1-P to fructose-6-P (Figure 1). The *mtlD* mutant suvB24 (SH1000 *mtlD*::Tn*917*) selected for further study showed clearly reduced survival on linoleic acid agar compared to its isogenic parent strain (Figure 2). Transduction of suvB24 into *S. aureus* Newman (Liv772; Table 1) identified a proportionately similar reduction in linoleic acid survival in this distinct strain background (data not shown).

### Culture Phenotypes of *mtl* Mutants

To investigate the role of the *mtlD* gene product in host cell physiology and to help explain the mechanism for reduced linoleic acid agar survival, growth of the suvB24 mutant was compared with its isogenic parental strain using a Biolog phenotype array (Biolog Inc. California, USA). Comparative growth arrays in the presence of various carbon, nitrogen, phophorous and sulphur compounds and a variety of amino acids, peptide nitrogen sources, osmolytes and pH ranges [28] identified that reduced Mtl metabolism was the only significantly altered phenotype (data not shown).

**Figure 3:**
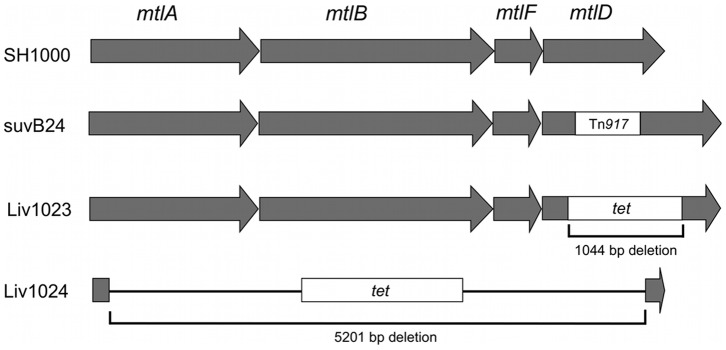
Schematic representation of the *mtlABFD* locus. Position of the transposon insertion and allelic replacements created during this study.

**Figure 4:**
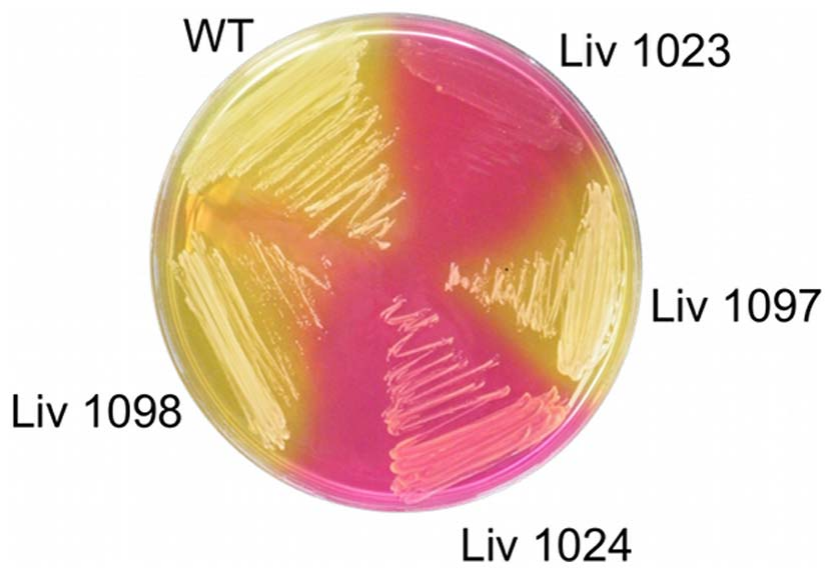
Mtl fermentation capability of *S. aureus* strains. Mtl fermentation is revealed by acid formation and colour change of the pH indicator to yellow. Liv1023 (SH1000 *mtlD*::*tet*) and Liv1024 (SH1000 *mtlABFD*::*tet*) do not ferment Mtl and this capability was restored by complementation with the entire locus in strains Liv1098 (SH1000 *mtlD*::*tet* pMJH71) and Liv1097 (SH1000 *mtlABFD*::*tet* pMJH71). Weak growth of Liv1023 was observed.

To confirm the role of the Mtl PTS operon in *S. aureus* cell survival, allelic replacement mutants were generated for *mtlD*, Liv1023 (SH1000 *mtlD*::*tet*) and for the entire *mtlABFD* operon, Liv1024 (SH1000 *mtlABFD*::*tet*) (Figure 3), using methods described previously [34], [35], [36]. Two complementation vectors were also generated by cloning the *mtlD* gene and the *mtlABFD* operon into the low copy shuttle vector pSK5632, producing plasmids pMJH70 and pMJH71, respectively. Cloning of the *mtlABFD* operon was achieved by transforming ligation products into strain Liv1021 (RN4220 *mtlD*::*tet*) selecting for fermentation on mannitol salt agar (MSA), since cloning of the operon in *E. coli* TOP10 was not successful, potentially due to toxicity. Complementation with *mtlD* alone did not restore Mtl fermentation on MSA due to the absence of a promoter for this distal gene; consequently complementation experiments were performed using pMJH71. Culture of Liv1023 (SH1000 *mtlD*::*tet*) and Liv1024 (SH1000 *mtlABFD*::*tet*) on MSA at 37°C demonstrated the inability of these mutants to ferment Mtl to produce acid (Figure 4). Weak growth was observed for Liv1023 on MSA agar in contrast to Liv1024, which grew similarly to the wild-type SH1000 strain. Metabolism was restored in the complemented strains Liv1097 (SH1000 *mtlABFD*::*tet* pMJH71) and LIV1098 (SH1000 *mtlD*::*tet* pMJH71) (Figure 4). Transduction of the *mtlD* and *mtlABFD* inactivations into *S. aureus* Newman (Liv1027 and Liv1028, respectively) confirmed the absence of Mtl fermentation in both mutants (data not shown).

**Figure 5:**
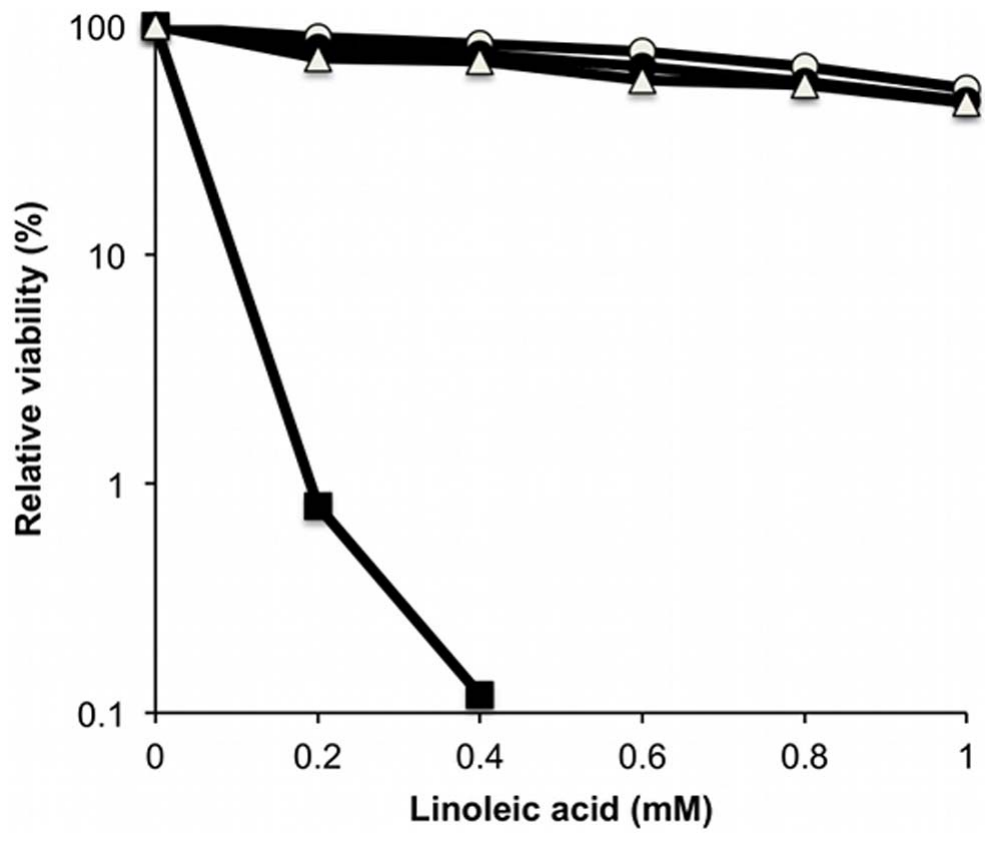
Survival on linoleic acid agar. Comparative survival of strains on BHI agar supplemented with 1 mM linoleic acid. Strains SH1000 (open circles), Liv1023 (SH1000 *mtlD*::*tet*) (filled squares), Liv1024 (SH1000 *mtlABFD*::*tet*) (open triangles) and Liv1098 (SH1000 *mtlD*::*tet* pMJH71) were diluted in PBS and equivalent volumes were plated onto the agar. SE from triplicate experiments is shown with error bars inside symbols.

Comparative growth assays of the allelic replacement mutants on linoleic acid agar confirmed that Liv1023 (SH1000 *mtlD*::*tet*) had an AFA growth defect similar to suvB24 (SH1000 *mtlD*::Tn*917*) with greater than 3-log reduction in survival (Figure 5). Similarly reduced levels of survival were observed following growth on agar supplemented with millimolar concentrations of oleic acid (C_18∶1Δ9_) or sapienic acid (C_16∶1Δ6_) (data not shown) demonstrating that inactivation of *mtlD* caused reduced survival to multiple AFAs. Allelic replacement of the *mtlABFD* operon did not impair survival from AFAs, in contrast to inactivation of *mtlD* alone. Proportionately reduced AFA survival was observed with an *mtlD* but not an *mtlABFD* inactivation in *S. aureus* Newman (Liv1027 and Liv1028, respectively; Table 1) (data not shown). Reduced survival of the *mtlD* mutant was fully complemented with the entire *mtlABFD* operon present on pMJH71 using strain Liv1098 (SH1000 *mtlD*::*tet* pMJH71) (Figure 2). The reduced survival of Liv1023 (SH1000 *mtlD*::*tet*) on linoleic acid agar was supported with a significantly reduced linoleic acid MIC (0.45±0.02 mM) (p<0.004) in BHI medium, compared to SH1000 (0.9±0.04 mM), Liv1024 (0.69±0.02 mM) and Liv1098 (0.85±0.03 mM).

**Figure 6:**
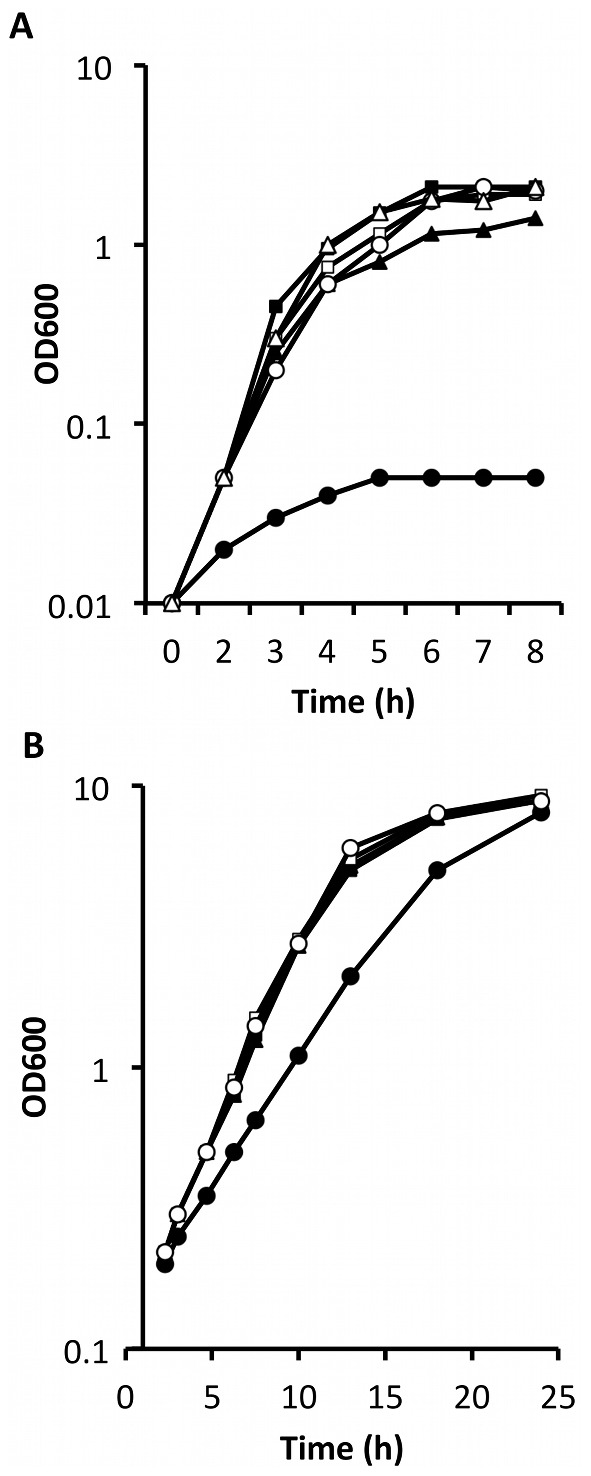
Growth phenotype of *mtlD* inactivated *S. aureus*. (A) Culture of strains in broth containing Mtl at 37°C. Liv1023 (SH1000 *mtlD::tet*) (•) had a significantly reduced growth rate (P<0.01, Student’s t-test) compared to wild-type (SH1000) (▪), Liv1024 (SH1000 *mtlABFD*::*tet*) (▴), strains Liv1098 (SH1000 *mtlD*::*tet* pMJH71) (□) and Liv1097 (SH1000 *mtlABFD*::*tet* pMJH71) (○). Calculated doubling times between 2 h and 3 h of growth: SH1000 = 0.33, Liv1023 (SH1000 *mtlD::tet*) = 1.7. Error bars indicate 1 SEM (n = 3). (B) Culture of strains in BHI broth at 25°C. Liv1023 (SH1000 *mtlD::tet*) (•) has a significantly reduced growth rate (P<0.001, Student’s t-test) compared to wild-type (SH1000) (▪), Liv1024 (SH1000 *mtlABFD*::*tet*) (▴), strains Liv1098 (SH1000 *mtlD*::*tet* pMJH71) (□) and Liv1097 (SH1000 *mtlABFD*::*tet* pMJH71) (○). Calculated doubling times between 5 h and 13 h of growth: SH1000 = 2.46, Liv1023 = 3.13. Representative dataset from triplicate assay.

Strain Liv1023 (SH1000 *mtlD*::*tet*) exhibited a profound growth defect when cultured in broth containing Mtl as the carbohydrate source (peptone 10 g l^−1^, Mtl 10 g l^−1^, beef extract 1 g l^−1^, NaCl 10 g l^−1^) (Figure 6A). Substituting the sugar alcohol Mtl for the sugars fructose or glucose restored normal growth, demonstrating the Mtl-specific defect (data not shown). *S. aureus* accumulates intracellular Mtl following incubation in the presence of glucose. To test if this accumulation affected survival from AFAs, the relative survival of exponential cells (OD_600_ = 1) of SH1000 incubated in PBS containing 1% (w/v) glucose was determined after growth on 1 mM linoleic acid agar. No clear difference in survival of the strains was observed.

**Figure 7:**
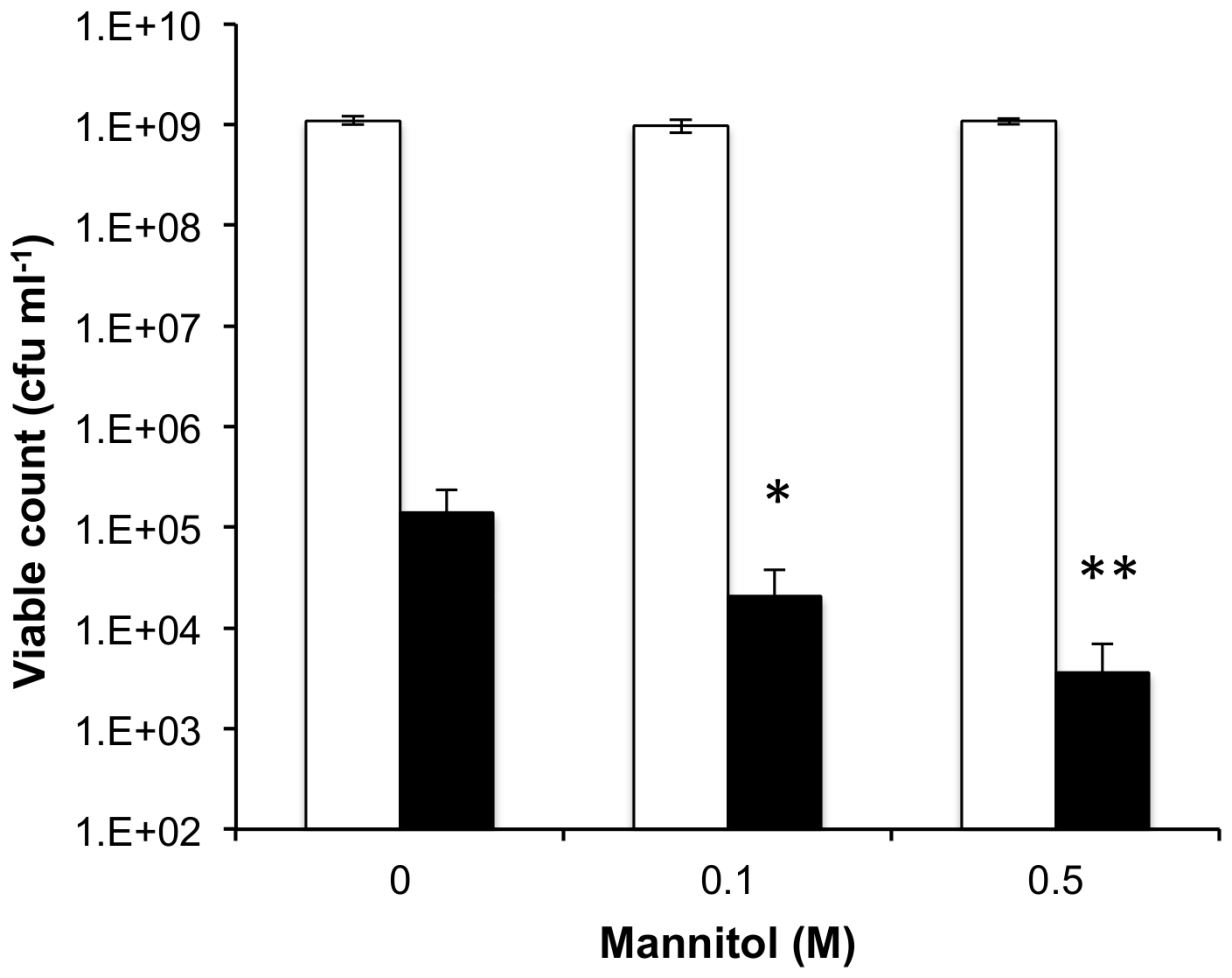
Growth of *S.* *aureus* in the presence of mannitol and linoleic acid. Bacteria were cultured on BHI agar containing either no or added mannitol (0.1 M, 0.5 M) in the presence (black bars) or absence (white bars) of 1 mM linoleic acid. Differences in viable cells recovered in the presence of mannitol were significantly reduced compared to the absence of mannitol (P = 0.03 and P = 0.001 for 0.1 M and 0.5 M, respectively).

All strains grew equally well at 37°C in BHI broth (data not shown), however a pronounced reduction in growth rate was observed for strain Liv1023 (SH1000 *mtlD*::*tet*) when cultured in BHI broth at 25°C (Figure 6B). This defect was specific to inactivation of *mtlD* but not for deletion of the complete operon. Starvation survival with limiting glucose was not impaired in *mtl* mutant strains [35], [37]. Growth of *S. aureus* SH1000 was tested in the absence or presence of mannitol (0.1 M, 0.5 M), with or without 1 mM linoleic acid to test for synergy. Mannitol was shown to have similar properties as ethanol [13], by acting synergistically with linoleic acid as evident by the reduced viable count with increasing mannitol concentration (Figure 7).

### Analysis of Cellular Metabolites

A comparative metabolomics analysis was undertaken to identify the intracellular metabolites of exponentially growing cells of strains SH1000, Liv1023 (SH1000 *mtlD*::*tet*) and Liv1024 (SH1000 *mtlABFD*::*tet*). This revealed that inactivation of *mtlD* resulted in an accumulation of Mtl and Mtl-P, the latter being undetectable in both SH1000 and Liv1024 (Table 2 and supplementary table 1). The total relative levels of Mtl species were over 20-fold greater in Liv1023 (SH1000 *mtlD*::*tet*) than SH1000. The near absence of Mtl in strain Liv1024 supports data that the MtlAB PTS transporter is the main portal for Mtl uptake [38]. Inactivation of *mtlD* and *mtlABFD* resulted in the absence of cellular Sorbitol-6-P (Table 2). Further clear differences in metabolite levels were evident in strain Liv1023 (*mtlD::tet*) relative to SH1000 and Liv1024 (Table S1).

**Table 2:** Sugar alcohols present in *S. aureus* strains.

	Relative mean concentration		
Metabolite	SH1000	Liv1023	Liv1024
Arabitol	116.1 (3.9)	107.5 (19.7)	51.4 (7.9)
Mannitol	417.6 (29.5)	1351.4 (82.5)	5.9 (0.9)
Mannitol-P	ND	8161.3 (119.7	ND
Ribitol	240 (22.3)	214.8 (17.6)	272.2 (27.1)
Sorbitol-6-P	149.4 (18.2)	ND	ND

GC-MS was used to analyse cytoplasmic fractions from exponential growth phase cells. 131 unique metabolites were compared and chromatograms and mass spectra were evaluated as described previously [8], [32] using the MSD ChemStation (Agilent, Palo Alto, CA, USA) and AMDIS (NIST, Gaithersburg, MD, USA) programs. The resulting data from triplicate samples (with less than 10% variability) were analyzed using a t-test. Samples with greater than 2-fold variation (p<0.05) were analyzed using the MetPA enrichment pathway analysis web application (http://metpa.metabolomics.ca/) [45]. ND, not detectable.

### Resistance and Cell Surface Properties of *mtl* Mutants

A range of antimicrobial agents were tested to determine if the observed reduced resistance of Liv1023 (SH1000 *mtlD*::*tet*) extended beyond AFAs. Growth and MICs were comparable between Liv1023 (SH1000 *mtlD*::*tet*) and SH1000 in the presence of a range of concentrations of NaCl, lauroyl sarcosine, SDS, dichlorophenyl and the human cathelicidin LL37 (Sigma). Liv1023 (SH1000 *mtlD*::*tet*) was observed to exhibit a lower MIC for H_2_O_2_ (1 mM) compared to SH1000 (4 mM) and Liv1024 (SH1000 *mtlABFD*::*tet*) (4 mM).

The hydrophobicity and zeta potential of all of the strains was similar when tested using either hexadecane partitioning or measured using a zetasizer (Malvern, UK), respectively (data not shown). The levels of carotenoid in cell membranes were similar between SH1000 and the *mtl* mutants, as judged by spectrophotometric analysis of methanol-extracted cells from overnight and 2 day-old cultures (data not shown).

### Virulence of *mtlD* Mutant

**Figure 8:**
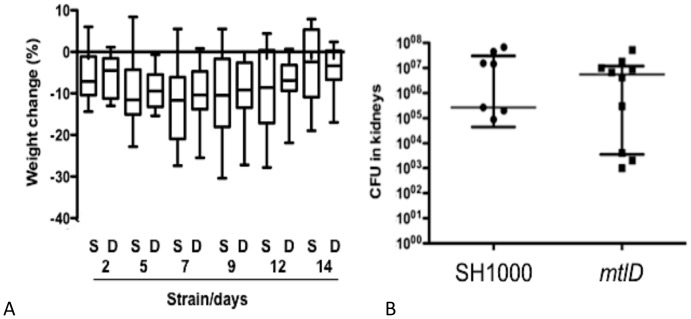
Virulence of *mtlD* in a murine infection. (A) Effect of WT SH1000 or Liv1023 (SH1000 *mtlD::tet*) on percentage change in weight of infected mice. There were no significant differences using Dunn’s test. (B) Effect of mutations of *mtlD* on cfu of *S. aureu*s SH1000 in kidneys of infected mice. There were no significant differences using the Mann Whitney Test.

The decreased *in vitro* AFA survival and reduced H_2_O_2_ MIC of the *mtlD* mutant prompted testing of its virulence compared to the isogenic parent strain using a previously described model of experimental septic arthritis (Figure 8). This model was tested to determine whether inactivation of the *mtlABFD* locus affected virulence, since its contribution to metabolism *in vivo* is unknown and the model generates abscesses where AFAs accumulate [14]. This revealed that SH1000 *mtlD* did not have reduced virulence, at least under the conditions studied [6], [32], [33].

## Discussion

The intrinsic importance of *S. aureus* carriage and transmission in relation to disease and its hypothesized link with virulence [39] requires that determinants are identified and characterised that promote survival in its primary niche and during its transient residence on human skin. From the study of gene mutants *S. aureus* defence from AFAs is achieved via a variety of surface components (IsdA, WTA, SasF) and regulation of peptidoglycan biosynthesis (VraRS, VraE), where a reduction in hydrophobicity to minimize access of the AFA to the membrane explains the contribution of several of these components to survival [6], [7], [19]. In addition, the arginine deiminase pathway increases survival [6], where its various contributions to metabolic versatility and its potential to modify local pH could explain its role.

Determining that an Mtl-1-P-dehydrogenase mutant, but not an *mtlABFD* transport operon mutant, has greatly reduced survival from AFAs implicates the accumulation of Mtl-1-P as being the causative factor. As the most abundant natural hexitol, Mtl is a carbon source for staphylococci and the inducible oxidation of Mtl-1-P generates fructose-6-P for entry into the Embden-Meyerhoff and hexosemonophosphate glycolytic pathways [38], [40]. All strains of *S. aureus* accumulate Mtl, despite not all being capable of using it for metabolism during aerobic growth. In *S. aureus* the cellular accumulation of Mtl was identified in resting cells when incubated in glucose or cultured in media without added carbohydrate [38]. Mtl accumulation was proposed to enhance metabolic versatility in *S. aureus,* however its mechanistic role is incompletely understood [41]. Following stress, such as after exposure to AFAs, utilisation of the pathway for Mtl conversion to fructose-6-P would regenerate NADH, thereby alleviating the pressure upon regenerating reactions downstream of pyruvate. In our previous studies [6], exposure of *S. aureus* to linoleic acid caused downregulated transcription of the *mtlABFD* locus, which suggests, either that reduced levels of intracellular Mtl is a preferred metabolic state following exposure to AFAs, or that lower amounts of Mtl-1-P arising from metabolism (concomitantly regenerating NADH) limited induction of the operon. A potential explanation for the reduced AFA survival of the *mtlD* mutant is its reduced adaptive capacity due to an inability to metabolise Mtl. The near wild-type AFA survival of the *mtlABFD* operon mutant argues against this Mtl metabolism hypothesis, however, unless there is an alternative metabolic reserve. 3-phosphoglycerate could serve as just such an alternative metabolic source and substrate for regenerating NADH, and of note there is 3-fold reduced 3-PGA in the *mtlD* mutant.

Metabolite analysis of the *S. aureus mtlD* mutant, when compared to the *mtlABFD* transport mutant and the parental strain, revealed that 14% of the total Mtl that accumulated intracellularly was not phosphorylated. Since the EIIMtl mannitol transporter (encoded by *mtlA*) phosphorylates the imported Mtl and since the Mtl-1-P-dehydrogenase activity is ablated in the *mtlD* mutant, the conversion of Mtl-1-P to Mtl in the *mtlD* mutant is likely to arise from phosphotransferase reactions as described by Saier and Newman [42]. Alternatively, an undescribed phosphatase activity might account for the presence of Mtl. In *Lactobacillus plantarum* a hypothetical phosphatase activity of EIIMtl was proposed to explain the apearance of Mtl in engineered strains [43]. In the study of Mtl overproducing strains of *L. lactis* a Mtl-1-phosphatase activity was proposed to explain the presence of unphosphorylated Mtl, where *mtlA* was absent, and thus an EIIMtl activity, could not be present. Analysis of the metabolites of growing cells of the *S. aureus mtlD* mutant cultured in BHI broth, when compared to the *mtlABFD* transport mutant and the parental strain, revealed further differences aside from sugar alcohol content (Table S1). These metabolite changes e.g. aminoadipic acid, 3-phosphoglycerate, hydroxypentanoic acid, heptanoic acid and tetradecanoylglycerol, do not indicate a clearly defined mechanistic explanation for decreased AFA MIC.

Growth of the *mtlD* mutant was strongly retarded in media containing mannitol, highlighting the deleterious effects resulting from the likely unrestricted accumulation of Mtl/Mtl-1-P. A direct link between the intracellular accumulation of Mtl/Mtl-1-P and reduced resistance to AFAs in *S. aureus* currently lacks an evidence-based mechanism. However, several features of the *mtlD* phenotype could result from a membrane-associated effect. Alcohol has a well-described potentiating mechanism with respect to AFAs and their membrane activity [13], since it is capable of solubilising membrane lipids due to its polarity and lipophilicity. Intracellularly accumulated sugar alcohol, Mtl, might act similarly to potentiate AFA action, since it was demonstrated in this study that Mtl acted synergistically with linoleic acid when added externally in BHI agar. Two further phenotypes point towards a membrane-specific alteration in the mtlD mutant; the reduced growth rate that was oberved for the *mtlD* mutant at 25°C and the reduced MIC for H_2_O_2_ which did not result from differences in catalase specific activity (data not shown). A perturbation in peroxide permeability at the membrane is consistent with the reduced MICs observed and might arise via Mtl potentiating the linoleic acid by virtue of the polarity and lipophilicity of alcohols affecting diffusion across the membrane, but this was not investigated further. No differences were observed between the staphyloxanthin levels in methanol extracts of any of the strains, which might be expected if the intracellular accumulation of Mtl altered membrane fluidity (data not shown) [44]. Mtl is frequently included in membrane preparations as a stabilising entity, either through direct effects or via osmotic stabilisation. The expression of a bacterial *mtlD* in *Saccharomyces cerevisiae* was sufficient to generate mannitol which was proposed to act as an osmolyte and was sufficient to rescue the phenotypes of a glycerol deficient mutant, producing an increased resistance to high salt and H_2_O_2_ [45]. Mtl is also proposed to function as an osmoprotectant in cells of petunia as well as improving cold tolerance [46].

The observed phenotype of reduced survival in the presence of AFAs did not translate to a reduction in virulence in a murine arthritis model or reduced MIC levels to a range of other membrane-acting agents. Thus, the changes to cellular physiology in the *mtlD* mutant are discrete, at least in this model of infection tested and other disease models, such as skin survival, remain to be tested.

## Supporting Information

Table S1 **Metabolites present in** ***S. aureus*** **strains.** Metabolites were identified using GC-MS analysis of cytoplasmic fractions from exponential growth phase cells. 131 unique metabolites were compared and chromatograms and mass spectra were evaluated as described previously [8], [32] using the MSD ChemStation (Agilent, Palo Alto, CA, USA) and AMDIS (NIST, Gaithersburg, MD, USA) programs. The resulting data from triplicate samples (with less than 10% variability) were analyzed using a t-test. Samples with greater than 2-fold variation (p<0.05) were analyzed using the MetPA enrichment pathway analysis web application (http://metpa.metabolomics.ca/) [45]. ND, not detectable.
(XLSX)
